# Closed-loop insulin delivery in suboptimally controlled type 1 diabetes: a multicentre, 12-week randomised trial

**DOI:** 10.1016/S0140-6736(18)31947-0

**Published:** 2018-10-13

**Authors:** Martin Tauschmann, Hood Thabit, Lia Bally, Janet M Allen, Sara Hartnell, Malgorzata E Wilinska, Yue Ruan, Judy Sibayan, Craig Kollman, Peiyao Cheng, Roy W Beck, Carlo L Acerini, Mark L Evans, David B Dunger, Daniela Elleri, Fiona Campbell, Richard M Bergenstal, Amy Criego, Viral N Shah, Lalantha Leelarathna, Roman Hovorka, APCam11 Consortium, B Alvarado, C Ashanti, J Baggott, K Balakrishnan, N Barber, L Bath, S Beasley, C Beatson, S Borgman, S Bradshaw, B Bugielski, AB Carlson, E Collett, J Curtis, J Demmitt, D Donahue, J Exall, R Forshaw, J Hayes, S Heath, A Hellmann, V Huegel, J Hyatt, L James, H Joseph, P Joshee, W Konerza, J Lum, M Madden, T Martens, C McCarthy, M McDonald, V Mikityuk, H Miles, D Miller, W Mubita, C Murphy, B Olson, R Pad, N Patibandla, K Riding, A Shaju, LA Thomas, J Thomson, D White, S Yau, J Yong

**Affiliations:** a Wellcome Trust-MRC Institute of Metabolic Science, University of Cambridge, Cambridge, UK; b Department of Paediatrics, University of Cambridge, Cambridge, UK; c Department of Diabetes and Endocrinology, Cambridge University Hospitals NHS Foundation Trust, Cambridge, UK; d Manchester University NHS Foundation Trust and University of Manchester, Manchester, UK; e Jaeb Center for Health Research, Tampa, FL, USA; f Royal Hospital for Sick Children, Edinburgh, UK; g Leeds Children's Hospital, Leeds, UK; h International Diabetes Center, Minneapolis, MN, USA; i Barbara Davis Center for Diabetes, University of Colorado Anschutz Medical Campus, Aurora, CO, USA

## Abstract

**Background:**

The achievement of glycaemic control remains challenging for patients with type 1 diabetes. We assessed the effectiveness of day-and-night hybrid closed-loop insulin delivery compared with sensor-augmented pump therapy in people with suboptimally controlled type 1 diabetes aged 6 years and older.

**Methods:**

In this open-label, multicentre, multinational, single-period, parallel randomised controlled trial, participants were recruited from diabetes outpatient clinics at four hospitals in the UK and two centres in the USA. We randomly assigned participants with type 1 diabetes aged 6 years and older treated with insulin pump and with suboptimal glycaemic control (glycated haemoglobin [HbA_1c_] 7·5–10·0%) to receive either hybrid closed-loop therapy or sensor-augmented pump therapy over 12 weeks of free living. Training on study insulin pump and continuous glucose monitoring took place over a 4-week run-in period. Eligible subjects were randomly assigned using central randomisation software. Allocation to the two study groups was unblinded, and randomisation was stratified within centre by low (<8·5%) or high (≥8·5%) HbA_1c_. The primary endpoint was the proportion of time that glucose concentration was within the target range of 3·9–10·0 mmol/L at 12 weeks post randomisation. Analyses of primary outcome and safety measures were done in all randomised patients. The trial is registered with ClinicalTrials.gov, number NCT02523131, and is closed to accrual.

**Findings:**

From May 12, 2016, to Nov 17, 2017, 114 individuals were screened, and 86 eligible patients were randomly assigned to receive hybrid closed-loop therapy (n=46) or sensor-augmented pump therapy (n=40; control group). The proportion of time that glucose concentration was within the target range was significantly higher in the closed-loop group (65%, SD 8) compared with the control group (54%, SD 9; mean difference in change 10·8 percentage points, 95% CI 8·2 to 13·5; p<0·0001). In the closed-loop group, HbA_1c_ was reduced from a screening value of 8·3% (SD 0·6) to 8·0% (SD 0·6) after the 4-week run-in, and to 7·4% (SD 0·6) after the 12-week intervention period. In the control group, the HbA_1c_ values were 8·2% (SD 0·5) at screening, 7·8% (SD 0·6) after run-in, and 7·7% (SD 0·5) after intervention; reductions in HbA_1c_ percentages were significantly greater in the closed-loop group compared with the control group (mean difference in change 0·36%, 95% CI 0·19 to 0·53; p<0·0001). The time spent with glucose concentrations below 3·9 mmol/L (mean difference in change −0·83 percentage points, −1·40 to −0·16; p=0·0013) and above 10·0 mmol/L (mean difference in change −10·3 percentage points, −13·2 to −7·5; p<0·0001) was shorter in the closed-loop group than the control group. The coefficient of variation of sensor-measured glucose was not different between interventions (mean difference in change −0·4%, 95% CI −1·4% to 0·7%; p=0·50). Similarly, total daily insulin dose was not different (mean difference in change 0·031 U/kg per day, 95% CI −0·005 to 0·067; p=0·09) and bodyweight did not differ (mean difference in change 0·68 kg, 95% CI −0·34 to 1·69; p=0·19). No severe hypoglycaemia occurred. One diabetic ketoacidosis occurred in the closed-loop group due to infusion set failure. Two participants in each study group had significant hyperglycaemia, and there were 13 other adverse events in the closed-loop group and three in the control group.

**Interpretation:**

Hybrid closed-loop insulin delivery improves glucose control while reducing the risk of hypoglycaemia across a wide age range in patients with suboptimally controlled type 1 diabetes.

**Funding:**

JDRF, NIHR, and Wellcome Trust.

## Introduction

Type 1 diabetes represents 5–10% of cases with diabetes worldwide, and is presently incurable.^1^ Achievement of recommended glycaemic control remains challenging across all age groups,^2^ in part because tight glycaemic control increases the risk of hypoglycaemia.^3^, ^4^

Over the past decade, considerable progress has been made in the development of closed-loop insulin delivery systems (the artificial pancreas), which couple continuous glucose monitoring and algorithm-directed insulin pump delivery.^5^ Hybrid closed-loop systems are characterised by automated insulin delivery, apart from when the user administers insulin boosts at meal time. In 2017, the first hybrid closed-loop system entered clinical use on the basis of a pivotal safety non-randomised, single-arm trial of a hybrid closed-loop system in patients with type 1 diabetes.^6^

**Research in context**

**Evidence before this study**

We searched PubMed for articles published up to June 12, 2018, using the terms (“artificial pancreas” OR “closed-loop”) AND (“type 1 diabetes mellitus” OR “diabetes”) AND (“outpatient” OR “home”) AND (“randomised” OR “randomised controlled trial”), for reports of randomised controlled trials published in English only. We identified 27 randomised trials that tested automated or semiautomated glucose control outside hospital settings. Outpatient use of automated insulin delivery systems is associated with an increased percentage of time during which sensor glucose is within the near normoglycaemic range, and reduced hyperglycaemia and hypoglycaemia, while modestly reducing glycated haemoglobin (HbA_1c_) in studies that were of long enough duration to report results for HbA_1c_. 13 of the 27 trials assessed day-and-night use of closed-loop systems. Seven of these 13 trials tested insulin-only systems, of which one trial assessed long-term use (≥12 weeks) of 24 h per day, 7 days per week, closed-loop. However, this study was done in adults only, and the participant number was small (n=33).

**Added value of this study**

To our knowledge, this multinational, multicentre study is the largest randomised study of closed-loop use in outpatient settings so far. It is also the longest randomised outpatient study of 24 h per day, 7 days per week, closed-loop use in children as young as 6 years and older. We showed that compared with sensor-augmented insulin pump therapy, day-and-night hybrid closed-loop insulin delivery significantly improved the percentage of time spent within the glucose target range (3·9–10·0 mmol/L) and mean glucose concentrations, and led to a significant decrease in HbA_1c_ while reducing hyperglycaemia and hypoglycaemia in a mixed population with suboptimally controlled type 1 diabetes. These improvements were seen irrespective of age.

**Implications of all the available evidence**

The use of day-and-night hybrid closed-loop insulin delivery improves glycaemic control while reducing the risk of hypoglycaemia in adults, adolescents, and children with type 1 diabetes compared with conventional pump therapy or sensor-augmented pump therapy. Results from our study together with those from previous studies support the adoption of closed-loop technology in clinical practice across all age groups.

Two meta-analyses of randomised trials reported that outpatient use of closed-loop systems increases the time sensor-measured glucose is near-normoglycaemia, and reduces the risk of hyperglycaemia and hypoglycaemia.^7^, ^8^ However, most trials had a small sample size, a short intervention period, and were done predominantly in adults. Only two studies^9^, ^10^ reported glycated haemoglobin [HbA_1c_] outcomes. This implies that effectiveness assessments from larger and appropriately designed and powered clinical trials are needed to support reimbursement and wider adoption of hybrid closed-loop systems.

In the present multicentre randomised trial, we hypothesised that the use of a hybrid closed-loop system improves glucose control and reduces the risk of hypoglycaemia compared with sensor-augmented pump therapy in individuals with suboptimally controlled type 1 diabetes. Hybrid closed-loop was applied over 12 weeks in a mixed population, including adults, adolescents, and children aged 6 years and older. We studied people with suboptimally controlled type 1 diabetes because we anticipated that this population might accrue particular benefits subject to satisfactory compliance and regular closed-loop use.

## Methods

### Study design

The study had an open-label, multicentre, multinational (the UK and the USA), randomised, parallel design. Insulin was delivered by contrasting day-and-night hybrid closed-loop (closed-loop group) or sensor-augmented pump therapy (control group) during free living over 12 weeks.^11^ Participants were recruited from diabetes outpatient clinics at four hospitals in the UK and two centres in the USA (see below).

Before study initialisation, approval was received from an independent research ethics committee in the UK (East of England–Cambridge East Research Ethics Committee), independent review boards in the USA (Jaeb Center for Health Research Institutional Review Board), regulatory authorities in the UK (Medicines and Healthcare products Regulatory Agency) and in the USA (Food and Drug Administration). Safety aspects were overseen by an independent data safety monitoring board. The study protocol is available online.

### Participants

Inclusion criteria included type 1 diabetes, as defined by WHO,^12^ for at least 1 year, insulin pump therapy for at least 3 months, and HbA_1c_ between 7·5% and 10% (58–86 mmol/mol). Participants were aged 6 years or older, with an equal proportion of children and young adults aged between 6 years and 21 years, and adults aged 22 years and older. Key exclusion criteria included regular use of real-time continuous glucose monitoring in the preceding 3 months, history of one or more episodes of severe hypoglycaemia in the preceding 6 months, and substantially reduced hypoglycaemia awareness in participants aged 18 years and older, as defined by a Gold score of 5 or more.^13^ A complete list of all inclusion and exclusion criteria is provided in the appendix.

We identified eligible adults from diabetes clinics attending Addenbrooke's Hospital (Cambridge, UK), Manchester Royal Infirmary (Manchester, UK), International Diabetes Center at Park Nicollet (Minneapolis, MN, USA), and Barbara Davis Center for Diabetes (Aurora, CO, USA). Children and adolescents were recruited from paediatric diabetes centres at Addenbrooke's Hospital (Cambridge, UK), Royal Hospital for Sick Children (Edinburgh, UK), Leeds Teaching Hospital (Leeds, UK), and International Diabetes Center at Park Nicollet, (Minneapolis, MN, USA). Eligible children and adolescents were identified by clinical teams at each centre, and were recruited by member of the local study team.

Study participants aged 16 years or older in the UK, 18 years or older in the USA, and parents or guardians of participants aged 15 years or younger in the UK and 17 years or younger in the USA gave written informed consent; written assent was obtained from minors.

### Randomisation and masking

Eligible participants who met criteria after the run-in period (see below) were randomly assigned using central randomisation software (SAS, version 9.4) to the use of day-and-night hybrid closed-loop or sensor-augmented pump therapy. The randomisation was stratified within centre by low (<8·5%) or high (≥8·5%) HbA_1c_. Implicit randomisation by age applied, given that each centre recruited either children and young adults (6–21 years), or adults (≥22 years). PC (study statistician) generated the sequence, which was used by local research teams to enrol and assign participants to the trial groups.

### Procedures

Participants in both study groups used a modified 640G insulin pump (investigational use only; Medtronic, Northridge, CA, USA), Enlite 3 glucose sensor (Medtronic), and Contour Next Link 2.4 glucometer (Ascensia Diabetes Care, Basel, Switzerland). Participants were not remotely monitored or supervised, and were able to do their usual activities. They were free to consume any meals of their choice and were allowed to participate in any indoor or outdoor physical activity. Participants were required to be present at regular visit intervals to receive appropriate training, and to be contactable via phone or email for scheduled study contacts to review device use. Data from the study insulin pump and glucometer were downloaded once per week by participants using Carelink software and stored on Carelink Clinical server (Medtronic). Blood samples were drawn for HbA_1c_ measurements at the hospitals where the patients were enrolled by qualified members of the centre's study team. Blood samples were taken at baseline, and at the start and at the end of the respective intervention period (closed-loop intervention or control intervention). An age-appropriate Pedatric Quality of Life Inventory (PedsQL) questionnaire was administered to participants (participant version) and guardians of participants aged 17 years and younger (parent proxy version) before and after the intervention period.

After training on the study pump and continuous glucose monitoring, participants underwent a run-in period of at least 4 weeks. During this period, participants were contacted once per week. Data obtained during this period could be used for adjustment of the insulin therapy. At the end of the run-in period, adherence to the use of study pump and continuous glucose monitoring was assessed. Before being randomly assigned to treatment, participants were required to show use of continuous glucose monitoring for at least 12 days, and use of the bolus calculator for at least 75% of meal boluses in the 2 weeks before randomisation.

Participants randomly assigned to the closed-loop group attended the clinical research facility for a 2–3-h visit. Training was provided on initiation and discontinuation of the hybrid closed-loop system, switching between closed-loop and standard insulin pump therapy, meal bolus procedure, and the use of study devices during exercise. Competency on the use of the closed-loop system was assessed. After discharge, participants applied the closed-loop system for the following 12 weeks. Participants randomly assigned to the control group (sensor-augmented insulin pump therapy) received additional training on the effective use of real-time continuous glucose monitoring for optimisation of insulin therapy. Participants were instructed not to activate the pump's threshold suspend or predictive low glucose features. Participants were free to optimise their treatment independently or on advice from health-care professionals.

Hypoglycaemia and hyperglycaemia alarms were activated according to personal preference and requirements in both study groups. The participants in both study groups had an identical number of planned contacts with the local study team. Participants were contacted within 24–48 h after the initiation of study treatment. During the first 2 weeks of the intervention, participants in the UK were contacted by phone or email, and those in the USA were seen in the clinic once per week. Thereafter, participants were contacted once per month. All participants were provided with a 24-h helpline to contact the study team in the event of study-related issues.

The closed-loop system (appendix) used a model predictive control algorithm (version 0.3.46, University of Cambridge, Cambridge, UK) on a smartphone (Galaxy S4, Samsung, Seoul, South Korea). Every 10 min, the control algorithm calculated an insulin infusion rate, which was set on the study pump. The control algorithm was initialised using preprogrammed basal insulin delivery downloaded from the study pump. Information about the participant's bodyweight and total daily insulin dose were entered at set-up. The treat-to-target control algorithm aimed to achieve glucose concentrations between 5·8 mmol/L and 7·3 mmol/L, depending on the accuracy of model-based glucose predictions.

The threshold suspend feature on the modified 640G pump was turned on during closed-loop operation and allowed insulin delivery to be suspended even when the smartphone was not within range or not operational. Further safety mitigations during closed-loop are detailed in the appendix.

HbA_1c_ was measured locally at screening, and at a central laboratory (University of Minnesota, Minneapolis, MN, USA) at the beginning and end of study interventions by use of an International Federation of Clinical Chemistry and Laboratory Medicine aligned method (Tosoh HPLC Glycohemoglobin Analyzer, Tosoh Medics, CA, USA; coefficient of variation range of 1·4–1·9%).

### Outcomes

The primary endpoint was the between-group difference in the proportion of time spent in the target glucose range of 3·9–10·0 mmol/L (70–180 mg/dL) based on sensor-measured glucose concentrations during the 12-week free-living phase.^14^ Secondary endpoints included HbA_1c_ concentration at 12 weeks; the mean (SD) and coefficient of variation of sensor-measured glucose concentrations over the 12-week study period; percentage of time with glucose concentrations in hypoglycaemia (<3·9 mmol/L, <3·5 mmol/L, and <2·8 mmol/L) and hyperglycaemic (>10·0 mmol/L and >16·7 mmol/L); the area under the curve below 3·5 mmol/L; insulin requirements (total, basal, and bolus); bodyweight; and participant and parent PedsQL score. A subset of endpoints, to restrict multiple comparisons, including the proportion of time spent in the glucose target range of 3·9–10·0 mmol/L, the percentage of time with glucose concentrations of less than 3·5 mmol/L, and the mean (SD) sensor-measured glucose concentration, was assessed during the day (0800 h to 2359 h) and night (2400 h to 0759 h). The utility analysis assessed the amount of sensor-measured glucose use in both study groups, and the amount of closed-loop system use in the closed-loop group.

The safety analysis assessed the frequency of severe hypoglycaemic episodes, frequency of severe hyperglycaemia (capillary blood glucose >16·7 mmol/L) with substantial ketosis (plasma ketones >0·6 mmol/L), and nature and severity of other adverse events, including diabetic ketoacidosis.

### Statistical analysis

On the basis of previous day-and-night closed-loop studies,^9^, ^15^ and an estimate of 10 percentage points (SD 14·5) improvement in time when glucose is within target range, 76 participants were required to achieve 85% power and an α level of 0·05 (two-tailed *t* test). 84 participants were planned to be randomly assigned to allow for dropouts.

Statistical analyses were done on an intention-to-treat basis. Mean and SD were reported for the primary and secondary outcomes, which had approximately normal distribution. For outcomes with skewed distribution, median and IQR are reported. For the primary outcome and secondary outcomes, the treatment group differences were analysed using linear models, while adjusting for HbA_1c_ at treatment initiation, corresponding run-in values for the study outcomes (ie, adjusting time spent in target glucose range during run-in when comparing the primary endpoint), and a random site effect. For comparison of bodyweight at 12 weeks, age and sex were additionally adjusted in the linear model. Normality of the residuals was assessed; if the residuals had highly skewed distribution, then ranked normal score transformation of outcome data was applied in the regression model and point estimate with 95% CI was constructed on the basis of the rank test.^16^ A per-protocol analysis was limited to participants with sensor glucose data availability for at least 50% of the time over the 12-week study period (both groups) and closed-loop use for at least 80% of the time when sensor glucose data were available (closed-loop group). All p values reported are two-sided, and no formal adjustments for multiple comparisons were made. A 5% significance level was used to declare statistical significance for the primary endpoint. Among the secondary endpoints, p values of less than 0·05 were used to define statistical significance for HbA_1c_, coefficient of variation of sensor glucose, percentage of time sensor glucose was below 3·9 mmol/L, percentage of time sensor glucose was above 10·0 mmol/L, total daily insulin, and bodyweight. For all other endpoints, statistical significance was defined at p values of less than 0·01. Separate p values for day and night glucose metrics were only calculated when the 24-h version of the same metric was statistically significant based on the above criteria. No formal statistical comparisons were made for safety outcomes (diabetic ketoacidosis and severe hyperglycaemia events) because of the small number of events. Outcomes were calculated using GStat, version 2.2.4, and statistical analyses were done using SAS, version 9.4.

This study is registered with ClinicalTrials.gov, number NCT02523131.

### Role of the funding source

The funders of the study had no role in the study design, data collection, data analysis, data interpretation, or writing of the report. Medtronic employees read the manuscript before submission as a courtesy. No changes were made in the manuscript following the review. The corresponding author had full access to all the data in the study and had final responsibility for the decision to submit for publication.

## Results

**Figure 1:**
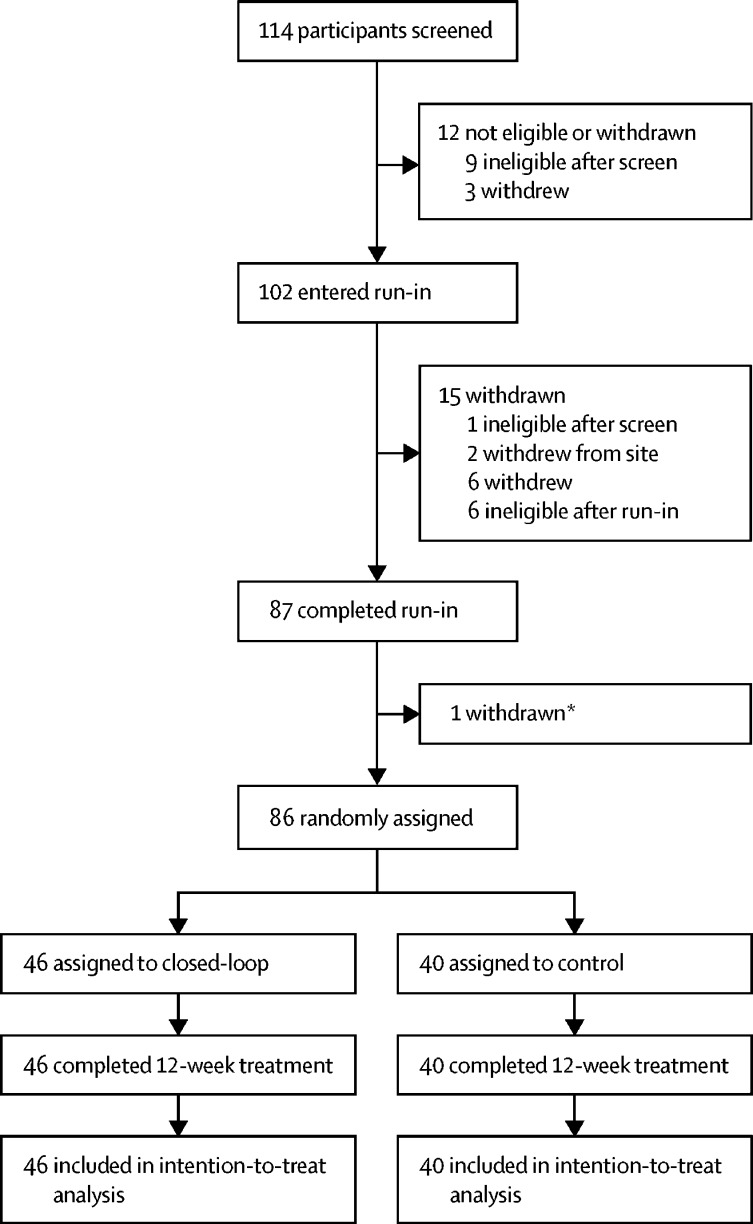
Trial profile *One patient had two severe hypoglycaemia events during run-in.

From May 12, 2016, to Nov 17, 2017, 114 individuals were screened. Nine participants did not meet inclusion criteria following screening assessment, and three withdrew before entering the run-in period. Another 15 participants did not successfully complete the run-in period. One participant was withdrawn after run-in because of non-compliance. 86 eligible participants were randomly assigned to treatment (figure 1). 46 participants were assigned to the closed-loop group and 40 participants to the control group. Of those enrolled, 44 participants were aged 22 years or older, 19 were aged 13–21 years, and 33 were aged 6–12 years.

Baseline characteristics are summarised in table 1 (breakdown in the appendix). After the run-in period, no participant failed the competency assessment and no participant withdrew post randomisation.

**Table 1:** Characteristics of the study participants at screening

		**Closed-loop (n=46)**	**Control (n=40)**
Sex			
	Female	22 (48%)	22 (55%)
	Male	24 (52%)	18 (45%)
Age, years		22 (13–36)	21 (11–36)
Age subgroup, years			
	6–12	11 (24%)	12 (30%)
	13–21	11 (24%)	8 (20%)
	22–39	18 (39%)	14 (35%)
	≥40	6 (13%)	6 (15%)
BMI* for age 20 years or older		28 (4), 24	27 (3), 21
BMI z score† for age 20 years or younger		0·70 (0·92), 22	0·69 (0·86), 19
Duration of diabetes‡		13 (7–20)	10 (7–19)
Total insulin dose, U/kg per day		0·76 (0·25)	0·69 (0·18)
Glycated haemoglobin at screening			
	Percentage	8·3% (0·6)	8·2% (0·5)
	mmol/mol of non-glycated haemoglobin	68 (7)	66 (6)

Data are n (%); median (IQR); mean (SD), n; mean (SD).

* Body-mass index (BMI) measured as kg/m^2^.

† BMI Z score adjusted for age and sex on the basis of 2000 CDC growth charts.

‡ Minimum duration of disease was 1·3 years, and maximum 45·6 years.

**Figure 2:**
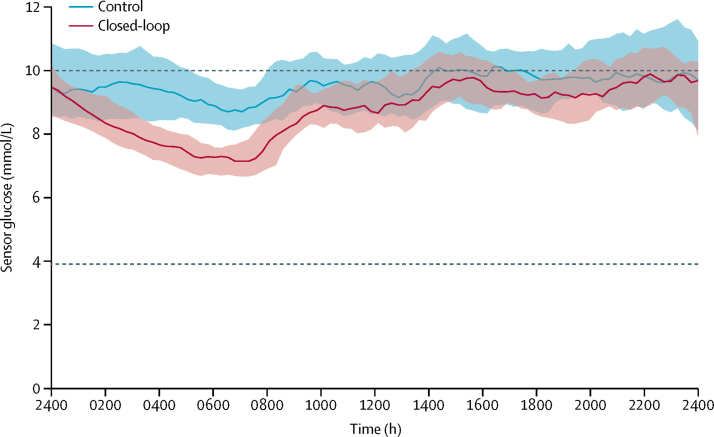
Sensor glucose Median (IQR) concentrations in the closed-loop group (red line and shaded area; n=46) and the control group (blue line and shaded area; n=40) are shown. Dashed lines indicate the target glucose range (3·9–10·0 mmol/L).

**Figure 3:**
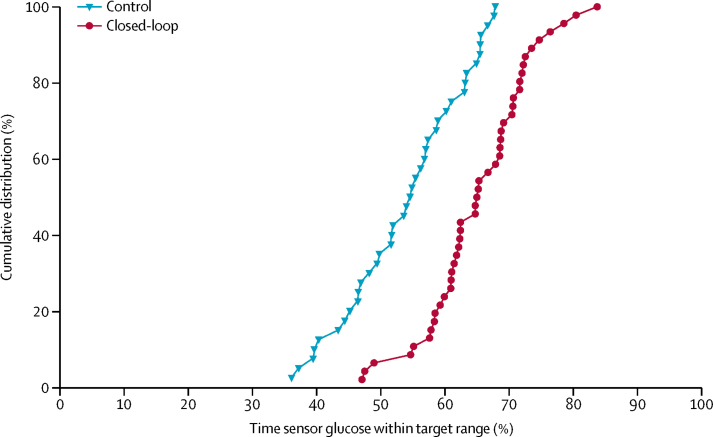
Cumulative distribution of percentage of time that sensor glucose was within the target range (ie, 3·9–10·0 mmol/L) over 12-week intervention phase by treatment group

Primary and secondary endpoints are summarised in table 2. 24-h sensor glucose profiles are shown in figure 2. The primary endpoint, the proportion of time sensor glucose was within the target range of 3·9–10·0 mmol/L, was 10·8 percentage points higher (95% CI 8·2–13·5; p<0·0001) in the closed-loop group (65%, SD 8) than in the control group (54%, SD 9). Improvements in time within target range were present in all three age groups (<13 years, 13–21 years, ≥22 years), in both sexes, and for both high and low baseline HbA_1c_ (appendix), with the majority of greater improvements present in participants with high baseline HbA_1c_ (post-hoc analysis, appendix). All participants in the closed-loop group had an improvement in percentage of time spent with glucose concentrations in target range compared with run-in period (appendix). A consistent difference of 10–15 percentage points occurred between the two groups across the whole range of time in range values, and a difference of nearly 20 percentage points among users with the highest time in range in the two groups (figure 3).

**Table 2:** Comparison of day-and-night glucose control during closed-loop and control periods

		**Baseline**		**12 weeks**		Difference (95% CI)*	p value*
		Closed-loop (n=46)	Control (n=40)	Closed-loop (n=46)	Control (n=40)		
Percentage of time with sensor glucose concentration in range							
	3·9 to 10·0 mmol/L†	52% (10)	52% (9)	65% (8)	54% (9)	10·8 (8·2 to 13·5)	<0·0001
	Less than 3·9 mmol/L	3·5% (2·0 to 5·4)	3·3% (1·2 to 5·5)	2·6% (1·9 to 3·6)	3·9% (1·7 to 5·3)	−0·83 (−1·40 to −0·16)‡	0·0130
	Less than 3·5 mmol/L	1·8% (0·8 to 3·2)	1·9% (0·6 to 3·3)	1·4% (0·9 to 1·9)	2·0% (0·9 to 3·0)	−0·33 (−0·81 to 0·04)‡	0·08
	Less than 2·8 mmol/L	0·4% (0·1 to 1·0)	0·5% (0·1 to 1·0)	0·3% (0·2 to 0·6)	0·5% (0·2 to 0·9)	−0·09 (−0·24 to 0·01)‡	0·11
	More than 10·0 mmol/L	44% (11)	44% (11)	32% (8)	42% (10)	−10·3 (−13·2 to −7·5)	<0·0001
	More than 16·7 mmol/L	5·5% (3·3 to 8·3)	4·9% (2·7 to 7·3)	3·5% (1·9 to 4·6)	4·4% (2·9 to 6·5)	−1·42 (−2·20 to −0·69)‡	<0·0001
Glycated haemoglobin							
	Percentage	8·0% (0·6)	7·8% (0·6)	7·4% (0·6)	7·7% (0·5)	−0·36% (−0·53 to −0·19)	<0·0001
	mmol/mol of non-glycated haemoglobin	63 (7)	62 (6)	57 (7)	60 (6)	−4·0 (−5·8 to −2·2)	<0·0001
Glucose AUC less than 3·5 mmol/L§		11 (5 to 25)	12 (4 to 25)	9 (5 to 15)	13 (6 to 23)	−2·3 (−5·4 to 0·3)‡	0·08
Glucose, mmol/L		9·8 (1·1)	9·8 (1·1)	8·9 (0·7)	9·7 (1·0)	−0·82 (−1·06 to −0·57)	<0·0001
SD of sensor glucose, mmol/L		3·9 (0·5)	3·8 (0·5)	3·5 (0·5)	3·8 (0·5)	−0·35 (−0·48 to −0·22)	<0·0001
Coefficient of variation of sensor glucose		40% (5)	39% (5)	40% (4)	40% (4)	−0·4% (−1·4 to 0·7)	0·50
Total insulin, U/kg per day		0·75 (0·22)	0·70 (0·18)	0·81 (0·25)	0·71 (0·19)	0·031 (−0·005 to 0·067)	0·09
Total basal insulin, U/kg per day		0·32 (0·07)	0·31 (0·08)	0·46 (0·13)	0·32 (0·10)	0·124 (0·099 to 0·150)	<0·0001
Total bolus insulin, U/kg per day		0·43 (0·19)	0·39 (0·14)	0·34 (0·17)	0·39 (0·13)	−0·087 (−0·114 to −0·060)	<0·0001
Bodyweight change from screening, kg		NA	NA	2·2 (2·3)	1·4 (2·6)	0·68 (−0·34 to 1·69)	0·19
PedsQL total score (participant version)		74 (12)	76 (14)	76 (12)	77 (12)	−0·3 (−4·1 to 3·4)	0·85
PedsQL total score (parent version)		69 (14), n=22	70 (15), n=19	74 (13), n=21	72 (11), n=19	3·0 (−2·7 to 8·7)	0·29

Data are mean (SD) or median (IQR). NA=not applicable. PedsQL=Pediatric Quality of Life Inventory.

* Model adjusted for baseline HbA_1c_, baseline value of the metric and site as a random effect. Difference is closed-loop minus control.

† Primary endpoint.

‡ Point estimates and CIs for metrics with a skewed distribution constructed from the rank test.

§ The area under the curve (AUC) is for a glucose level of less than 3·5 mmol/L per 24-h period.

In both groups, HbA_1c_ concentration was reduced from screening (closed-loop 8·3% [SD 0·6], 68 mmol/mol [SD 7]; control 8·2% [SD 0·5], 66 mmol/mol [SD 5]) to post run-in assessment (closed-loop 8·0% [SD 0·6], 63 mmol/mol [SD 7]; control 7·8% [SD 0·6], 62 mmol/mol [SD 6]). HbA_1c_ concentrations were significantly lower after closed-loop intervention (7·4% [SD 0·6], 57 mmol/mol [SD 7]) compared with control intervention (7·7% [SD 0·5], 60 mmol/mol [SD 6]), with a mean difference between groups favouring the closed-loop group by 0·36% (95% CI 0·19–0·53; 4·0 mmol/mol, 95% CI 2·2–5·8; p<0·0001). HbA_1c_ improvements were not different among children, adolescents, and adults (appendix).

Day-and-night closed-loop therapy significantly reduced mean glucose (p<0·0001) and time spent above target (p<0·0001) compared with the control group. Glucose variability, measured as the SD of sensor glucose was lower in the closed-loop group than in the control group (p<0·0001). The coefficient of variation of sensor glucose was not different between groups (p=0·50).

Closed-loop therapy significantly reduced the percentage of time sensor glucose was below 3·9 mmol/L (p=0·0130). The percentage of time spent with sensor readings below 3·5 mmol/L and 2·8 mmol/L was low, and not different between interventions (table 2). The burden of hypoglycaemia, as measured by the area under the curve when sensor glucose was less than 3·5 mmol/L, was not different between the groups (p=0·08).

Increased time when glucose was within target range, reduced mean glucose, reduced time when glucose was below target, and a reduction in HbA_1c_ was achieved by the closed-loop group without increasing total daily insulin (p=0·09). Higher basal insulin delivery than the control group during closed-loop therapy (p<0·0001) was offset by lower bolus delivery (p<0·0001). The change in bodyweight from the screening value was not different between groups (closed-loop 2·2 [SD 2·3] *vs* control 1·4 [SD 2·6]; p=0·19). The use of the closed-loop system was not associated with any additional burden, as assessed by the participant version of PedsQL (p=0·85) or the parent version (p=0·29; table 2).

Benefits of closed-loop therapy were prominent during the night (table 3, figure 2). Closed-loop therapy significantly reduced daytime and night-time mean glucose and glucose variability (for both, daytime p=0·0003, night-time p<0·0001). The proportion of time when glucose was within the target range, between 3·9 mmol/L and 10·0 mmol/L, was significantly greater in the closed-loop group than the control group (day and night, both p<0·0001).

**Table 3:** Day-and-night glucose control during closed-loop and control periods

		**Baseline**		**12 weeks**		Difference (95% CI)*	**p value**
		Closed-loop (n=46)	Control (n=40)	Closed-loop (n=46)	Control (n=40)		
**Day (0800 h to 2359 h)**							
Percentage of time with sensor glucose level in range							
	3·9–10·0 mmol/L	52% (10)	51% (9)	59% (9)	53% (9)	5·9 (3·1 to 8·7)	<0·0001
	Less than 3·5 mmol/L	1·6% (0·9 to 2·7)	1·9% (0·8 to 3·3)	1·6% (0·9 to 2·1)	2·2% (0·9 to 2·8)	NA†	NA†
Glucose, mmol/L		10·0 (1·2)	9·9 (1·1)	9·3 (0·8)	9·8 (1·0)	−0·51 (−0·77 to −0·24)	0·0003
SD of sensor glucose, mmol/L		4·0 (0·6)	3·9 (0·5)	3·7 (0·5)	3·9 (0·5)	−0·26 (−0·40 to −0·12)	0·0003
**Night (2400 h to 0759 h)**							
Percentage of time with sensor glucose level in range							
	3·9–10·0 mmol/L	54% (13)	53% (14)	77% (8)	56% (13)	21·5 (17·9 to 25·0)	<0·0001
	Less than 3·5 mmol/L	1·8% (0·6 to 4·1)	1·8% (0·5 to 3·9)	1·0% (0·7 to 1·8)	2·2% (0·7 to 3·3)	NA†	NA†
Glucose, mmol/L		9·5 (1·4)	9·6 (1·5)	8·0 (0·7)	9·4 (1·2)	−1·46 (−1·76 to −1·16)	<0·0001
SD of sensor glucose, mmol/L		3·6 (0·5)	3·5 (0·5)	2·9 (0·5)	3·6 (0·5)	−0·67 (−0·84 to −0·49)	<0·0001

Data are mean (SD) or median (IQR).

* Difference is closed-loop minus control.

† p value not computed as 24-h result was not significantly different; thus, separate day and night comparisons were not done.

Day-and-night closed-loop therapy was used for a median of 71% (63–83) of the time over the 12-week period, and participants in the closed-loop group wore a glucose sensor for a median of 90% (83–95) of the time (appendix). Control group participants wore a glucose sensor over a median of 90% (81–95) of the time. The number of planned contacts (ie, visits, email, or phone calls) was the same in both groups. However, more unscheduled contacts took place in the closed-loop group than in the control group (n=69 *vs* n=17; appendix). In the closed-loop group, a greater number of threshold suspend events occurred during the day than the night (appendix).

In a prespecified per-protocol analysis of the primary endpoint, comprising 24 participants in the closed-loop group and 39 participants in the control group (appendix), similar results to those from the intention-to-treat analysis were observed (closed-loop 68% [SD 8] *vs* control 54% [SD 9]; p<0·0001; appendix). These results did not change when less stringent per-protocol criteria were used (post-hoc analysis, appendix).

Post randomisation, no severe hypoglycaemia occurred in either study group. One diabetic ketoacidosis presented in the closed-loop group due to infusion set failure, and was not related to the closed-loop therapy (table 4). Two participants in each study group had significant hyperglycaemia with capillary glucose greater than 16·7 mmol/L and elevated plasma ketones (>0·6 mmol/L). There were 13 other adverse events in the closed-loop group and three in the control group (appendix); all were unrelated to treatment. All participants recovered fully without clinical sequelae. Protocol deviations were comparable between study groups (appendix).

**Table 4:** Adverse events

		**Closed-loop (n=46)**	**Control (n=40)**
**Diabetic ketoacidosis**			
Number of events per participant			
	0	45	40
	1	1	0
Incidence rate, per 100 person-years		8·7	0
Number of participants with at least one diabetic ketoacidosis event		1 (2%)	0 (0%)
Severe hyperglycaemia*			
Number of events per participant			
	0	44	38
	1	2	2
Incidence rate, per 100 person-years		17·4	20·3
Number of participants with at least one severe hyperglycaemia event		2 (4%)	2 (5%)

Data are n or n (%), unless otherwise stated. There was no severe hypoglycaemia event and no other serious adverse event besides those reported above in either treatment group.

* Defined as capillary glucose concentration of more than 16·7 mmol/L (300 mg/dL) and plasma ketones of more than 0·6 mmol/L.

## Discussion

In this multinational, multicentre, open-label, randomised trial, we show that 12-week use of a day-and-night hybrid closed-loop insulin delivery system, compared with sensor-augmented insulin pump therapy, was associated with an improvement in overall glucose control and a reduction in hypoglycaemia risk in suboptimally controlled type 1 diabetes in children, adolescents, and adults. The hybrid closed-loop system was used safely during daily living without supervision or remote monitoring.

We report a 10·8 percentage point increase in time with glucose concentrations within the target glucose range across all age groups. This improvement resulted from a reduction of time spent in hyperglycaemia without change in total insulin delivery. We observed a lower amount of bolus insulin and a higher amount of basal insulin in the closed-loop group than in the control group. Lower bolus insulin requirements in the closed-loop group than in the control group could be explained by lower glucose concentrations in this group during closed-loop use, lessening the need for correction boluses. The insulin to carbohydrate ratio did not need to be increased, unlike in other closed-loop systems,^17^ simplifying clinical adoption of our closed-loop system. Benefits of the closed-loop were greater overnight because, even with the use of a closed-loop system, daytime control is typically confounded by meals and physical activity. These improvements are attributable to the use of the closed-loop system alone because no regular adjustments of insulin pump therapy driven by a health-care professional took place, unlike in another study.^17^

The findings of the present study are consistent with results from our previous trials during free living in children and adolescents,^9^ adults with well controlled type 1 diabetes,^18^ and adults with less well controlled type 1 diabetes.^9^ This consistency of findings underpins the robustness of our model predictive algorithm, and supports the application of our closed-loop systems across a wide range of people with type 1 diabetes.

Use of hybrid closed-loop therapy led to a modest, but clinically significant, 0·36% reduction in HbA_1c_, compared with sensor-augmented pump therapy. This reduction was additive to that observed during the run-in phase, the latter attributable to the observer bias and initiation of continuous glucose monitoring. The decrease in HbA_1c_ during closed-loop use was slightly greater than that observed in two randomised trials^9^, ^10^ run for long enough to assess changes in HbA_1c_, both adopting sensor-augmented pump therapy as a comparator. Thabit and colleagues^9^ showed a mean reduction in HbA_1c_ by 0·3% with day-and-night hybrid closed-loop therapy, whereas Kropff and colleagues^10^ reported a reduction of 0·2% for evening-and-night closed-loop application. These two trials were small, with approximately 30 participants per trial, and closed-loop application was restricted to adults. In comparison, the present study randomly assigned 86 participants and the age range was wider. Improvements in HbA_1c_ in the present study were consistent across all age groups.

The proportion of patients who experienced a hypoglycaemic event was low in the present study and comparable to other outpatient closed-loop studies.^9^, ^17^ The reduction in the proportion of time spent in hypoglycaemia below 3·9 mmol/L with closed-loop therapy was statistically significant; time below 3·5 mmol/L and 2·8 mmol/L did not reach statistical significance. Because no severe hypoglycaemia presented in either group, the effect of closed-loop therapy on severe hypoglycaemia remains unclear. Further reduction of hypoglycaemia risk might be achieved through the addition of glucagon in bihormonal closed-loop systems,^19^, ^20^ particularly during exercise.^21^, ^22^

The strengths of our study are the multicentre, multinational design and the wide age range of participants, which support generalisability of study findings. The study was done without remote monitoring or close supervision in free-living settings, allowing for real-world assessment of performance of closed-loop systems. No investigator-led optimisation of insulin therapy took place, and improvements in glucose outcomes with closed-loop therapy are solely attributable to its use. Limitations include the number of devices comprising our hybrid closed-loop system, which increased the risk of device and connectivity problems, and resulted in more frequent non-protocol contacts to address technical issues. Threshold suspend and predictive low glucose suspend features^4^, ^23^ were not enabled in the control group because the study objective was to compare algorithmic and non-algorithmic insulin delivery approaches. We excluded participants with HbA_1c_ outside the range of 7·5–10·0% and other groups, such as those with an impaired awareness of hypoglycaemia or a history of recurrent severe hypoglycaemia, although these subgroups might benefit from use of the closed-loop system.

In conclusion, we found that free-living use of hybrid closed-loop insulin delivery over a period of 12 weeks led to clinically meaningful improvements in glycaemic control, while reducing the risk of hypoglycaemia in suboptimally controlled type 1 diabetes in adults, adolescents, and children aged 6 years and older.

For the **study protocol** see https://www.mrl.ims.cam.ac.uk/wp-content/uploads/2018/09/APCam11-protocol-v5.1-2017_06_16-clean.pdf

**This online publication has been corrected. The corrected version first appeared at thelancet.com on October 11, 2018**

## Data sharing
