# Department of Error

**DOI:** 10.1016/S0140-6736(18)32477-2

**Published:** 2018-10-13

*Tauschmann M, Thabit H, Bally L, et al. Closed-loop insulin delivery in suboptimally controlled type 1 diabetes: a multicentre, 12-week randomised trial.* Lancet *2018; **392:** 1321–29*—In table 1 of this Article (published online Oct 3), the data presented for the total insulin dose, U/kg per day, should have been 0·76 (0·25) for the closed-loop group and 0·69 (0·18) for the control group. These corrections have been made to the online version as of Oct 11, 2018, and the printed Article is correct.

