# Supplementary material for: Closed-loop insulin delivery in suboptimally controlled type 1 diabetes: a multicentre, 12-week randomised trial
Source: Lancet. 2018 Oct 13;392(10155):1321–9. doi: 10.1016/S0140-6736(18)31947-0 (PMC6182127; doi:10.1016/S0140-6736(18)31947-0)
Supplement: Supplementary appendix [file mmc1.pdf]

THE LANCET

Supplementary appendix

This appendix formed part of the original submission and has been peer reviewed. We post it as supplied by the authors.

Supplement to: Tauschmann M, Thabit H, Bally L, et al. Closed-loop insulin delivery in suboptimally controlled type 1 diabetes: a multicentre, 12-week randomised trial. *Lancet* 2018; published online Oct 3. http://dx.doi.org/10.1016/S0140-6736(18)31947-0.

Supplementary Appendix

Table of contents

APCam11 Consortium

Inclusion and exclusion criteria

Safety precautions during closed-loop use

Figure S1. FlorenceM system

Figure S2. Scatter plot for percentage time glucose was in target range over 12 weeks

Table S1. Characteristics of the study participants at screening

Table S2. Sensor glucose time in range according to subgroups

Table S3. Selected outcomes by glycated haemoglobin stratum at screening

Table S4. Selected secondary endpoints according to age subgroups

Table S5. Comparison of sensor glucose use by treatment group

Table S6. Unscheduled contacts by treatment group

Table S7. Pump threshold suspend events in the closed-loop group

Table S8. Subjects excluded from per-protocol analysis

Table S9. Per-protocol analysis results (primary outcome)

Table S10. Per-protocol analysis results (primary outcome) using 70% closed-loop use as a cut-point

Table S11. Adverse events

Table S12. Protocol deviations by treatment group

APCam11 Consortium

The following investigators from the APCam11 Consortium contributed to the work:

University of Cambridge, UK: M Tauschmann, H Thabit, L Bally, JM Allen, ME Wilinska, Y Ruan, J Hayes, N Barber, J Curtis, CL Acerini, DB Dunger, ML Evans, R Hovorka

Cambridge University Hospitals NHS Foundation Trust, UK: S Hartnell

Central Manchester University Hospitals NHS Foundation Trust, UK: L Leelarathna, H Thabit, A Shaju, S Yau, L James, W Mubita, K Balakrishnan, D Donahue, W Mubita

Leeds Children's Hospital, UK: J Exall, R Forshaw, R Pad, S Heath, J Yong, F Campbell

Jaeb Center for Health Research, Tampa, FL, USA: J Sibayan, C Kollman, P Cheng, RW Beck, J Lum, S Borgman, V Huegel, S Bradshaw, A Hellman, N Patibandla, C McCarthy, C Murphy, B Bugielski, B Alvarado

Royal Hospital for Sick Children, Edinburgh, UK: D Elleri, M McDonald, L Bath, H Miles, D Miller, E Collett, J Baggott, K Riding, J Thomson

International Diabetes Center, Minneapolis, MN, USA: RM Bergenstal, A Criego, AB Carlson, T Martens, S Beasley, M Madden, B Olson, J Hyatt, C Ashanti, V Mikityuk, LA Thomas, W Konerza

Barbara Davis Center for Diabetes, University of Colorado Denver, CO, USA: VN Shah, H Joseph, C Beatson, J Demmitt, P Joshee, D White

Inclusion and exclusion criteria

Inclusion criteria

1. The subject is at least 6 years or older [with equal proportion of youth (6 to 21 years) and adults (22 years and older)]
2. The subject has type 1 diabetes, as defined by WHO for at least 1 year or is confirmed C-peptide negative
3. The subject will have been an insulin pump user for at least 3 months, with good knowledge of insulin self-adjustment as judged by the investigator
4. The subject is treated with one of the rapid acting insulin analogues (insulin Aspart, Lispro or Glulisine)
5. The subject is willing to perform regular capillary blood glucose monitoring, with at least 4 blood glucose measurements taken every day
6. Screening HbA1c ≥ 7.5% (58.5mmol/mol) and ≤ 10 % (86mmol/mol) based on analysis from local laboratory or equivalent [with equal proportion of subjects above and below HbA1c 8.5% (69mmol/mol)]
7. The subject is literate in English
8. The subject is willing to wear glucose sensor
9. The subject is willing to wear closed loop system at home
10. The subject is willing to follow study specific instructions
11. The subject is willing to upload pump and CGM data at regular intervals
12. The subject is willing to restrict alcohol consumption to ≤ 2 units per day throughout the study period
13. Female subjects of child bearing age should be on effective contraception and must have a negative urine-HCG pregnancy test at screening.
14. The subject lives with someone who is trained to administer intramuscular glucagon and is able to seek emergency assistance.
15. The subject has access to WIFi at home.

Exclusion criteria

1. Non-type 1 diabetes mellitus including those secondary to chronic disease
2. Subject using real-time CGM on regular basis in preceding 3 months
3. Any other physical or psychological disease likely to interfere with the normal conduct of the study and interpretation of the study results as judged by the investigator
4. Untreated coeliac disease or thyroid disease or subject is being treated for hypothyroidism at time of screening
5. Current treatment with drugs known to interfere with glucose metabolism, e.g. systemic corticosteroids, non-selective beta-blockers and MAO inhibitors etc.
6. Known or suspected allergy to insulin
7. Subjects with clinically significant nephropathy (eGFR < 45ml/min) or on dialysis, neuropathy or active retinopathy (defined as presence of maculopathy or proliferative changes) as judged by the investigator
8. Adults: one or more episodes of severe hypoglycaemia as defined by American Diabetes Association (33) in preceding 6 months; Youth: recurrent incidents of severe hypoglycaemia during the previous 6 months (Adults and adolescents: severe hypoglycaemia is defined as an event requiring assistance of another person to actively administer carbohydrates, glucagon, or take other corrective actions including episodes of hypoglycaemia severe enough to cause unconsciousness, seizures or attendance at hospital; children: severe hypoglycaemia is defined as an event associated with a seizure or loss of consciousness);
9. Random C-peptide > 100pmol/l with concomitant plasma glucose >4 mmol/l (72 mg/dl)
10. Regular use of acetaminophen
11. Lack of reliable telephone facility for contact
12. Total daily insulin dose ≥ 2 IU/kg/day
13. Total daily insulin dose < 15 IU/day
14. Pregnancy, planned pregnancy, or breast feeding
15. Severe visual impairment
16. Severe hearing impairment

17. Significantly reduced hypoglycaemia awareness in subjects 18 year and older (screening Gold score > 4)
18. Subjects using implanted internal pace-maker
19. Patients with medically documented allergy towards the adhesive (glue) of plasters or Subject is unable to tolerate tape adhesive in the area of sensor placement
20. Serious skin diseases (e.g. psoriasis vulgaris, bacterial skin diseases) located at places of the body, which potentially are possible to be used for localisation of the glucose sensor)
21. Subject is currently abusing illicit drugs
22. Subject is currently abusing prescription drugs
23. Subject is currently abusing alcohol
24. Subject is using pramlintide (Symlin) at time of screening
25. Subject has elective surgery planned that requires general anaesthesia during the course of the study
26. Subject is a shift worker with working hours between 10pm and 8am
27. Subject has a sickle cell disease, haemoglobinopathy; or has received red blood cell transfusion or erythropoietin within 3 months prior to time of screening
28. Subject plans to receive red blood cell transfusion or erythropoietin over the course of study participation
29. Subject diagnosed with current eating disorder such as anorexia or bulimia
30. Subject plans to use significant quantity of herbal preparations (use of over the counter herbal preparation for 30 consecutive days or longer period during the study) or significant quantity of vitamin supplements (four times the recommended daily allowance used for 30 consecutive days or longer period during the study) during the course of their participation in the study

Safety precautions during closed-loop use

Participants were trained to perform a glucose sensor calibration check before breakfast and evening meals. If sensor glucose was greater than capillary fingerstick glucose by more than 3·0mmol/l, the glucose sensor was recalibrated. If sensor glucose became unavailable or when communication between the smartphone and the study pump was interrupted, pre-programmed insulin delivery automatically restarted within 30 minutes. This limited the risk of insulin under- and over-delivery.

Safety rules limited the maximum insulin infusion and suspended insulin delivery of sensor glucose at or less than 4·3mmol/l or when sensor glucose was rapidly decreasing. The threshold suspend feature on 640G pump was turned on during closed-loop and allowed insulin delivery to be suspended if the smartphone was not in range. Resumption of insulin delivery followed the standard rules associated with threshold suspend. The threshold suspend sensor glucose value was set initially at 2.8mmol/l (50mg/dl), but could be modified by the participant as required.

Figure S1. FlorenceM closed-loop system prototype. The system consists of a continuous glucose monitoring transmitter with Enlite 3 sensor (Medtronic), an insulin pump (modified 640G pump, Medtronic), and a mobile phone (Galaxy S4, Samsung) running the control algorithm (Cambridge). The smartphone communicated wirelessly with the modified investigational-use-only 640G pump through a proprietary translator device included in the smartphone's enclosure. Every 10 min, the control algorithm received sensor and insulin delivery data from the pump, and calculated an insulin infusion rate which was set on the study insulin pump.

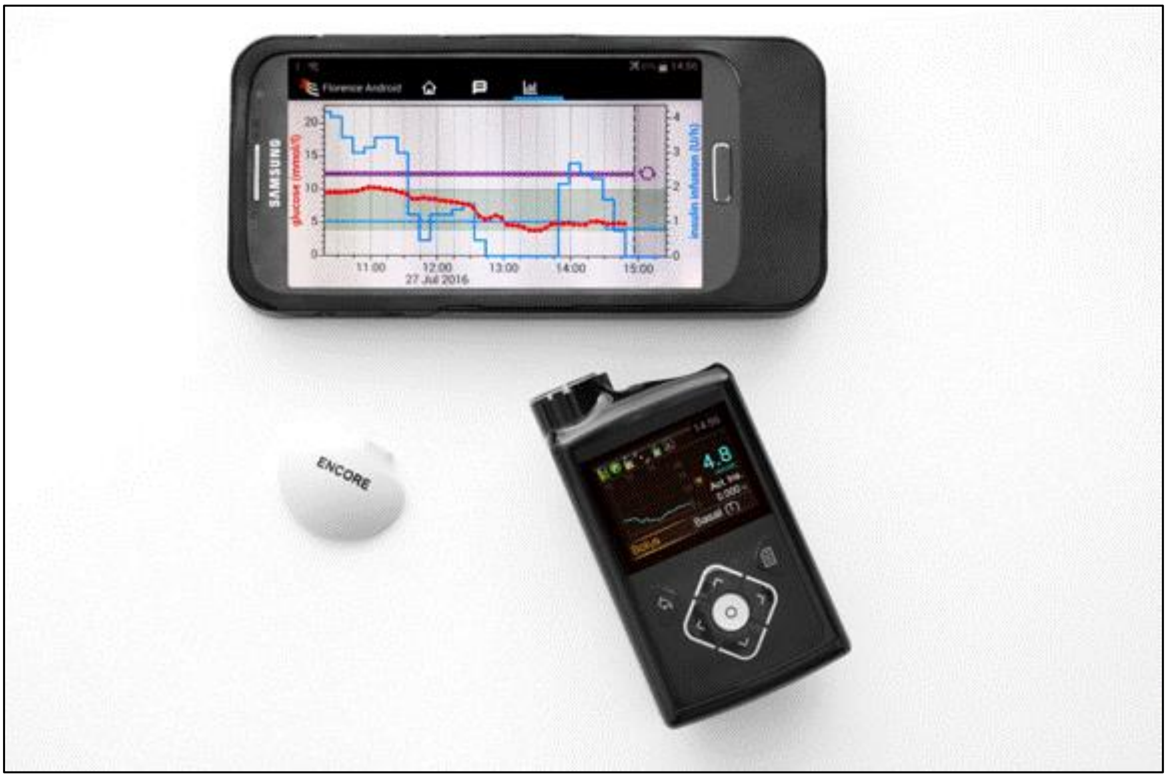

Figure S2. Scatter plot for percentage time that sensor glucose was in target range over 12-week intervention phase vs. baseline by treatment group (N=86).

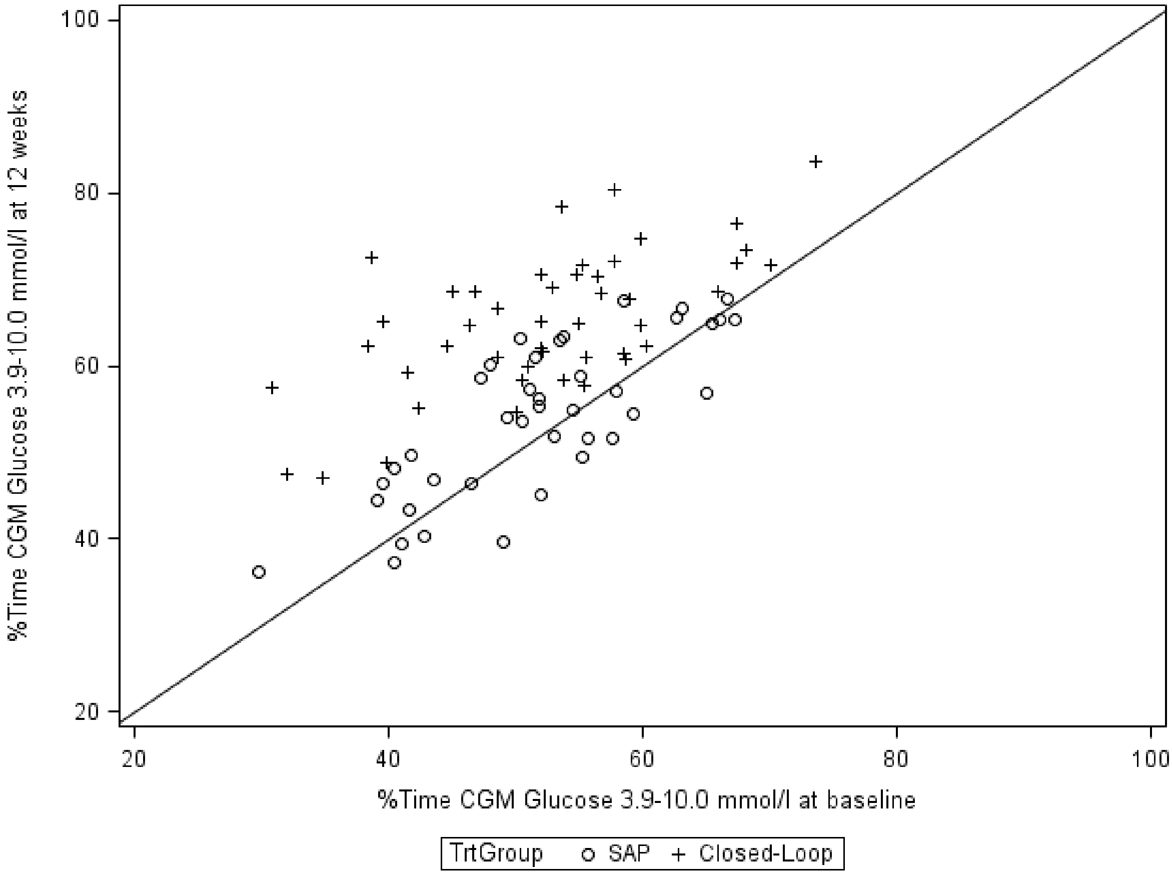

Table S1. Breakdown of characteristics of the study participants at screening.

	**Closed-loop (N=46)**	**Control (N=40)**
Age (yrs)		
6-<13	11 (24%)	12 (30%)
13-<22	11 (24%)	8 (20%)
22-<40	18 (39%)	14 (35%)
≥40	6 (13%)	6 (15%)
median (IQR)	22 (13 to 36)	21 (11 to 36)
range	8 - 65	6 - 60
Diabetes Duration (yrs)		
1-<5	8 (17%)	6 (15%)
5-<10	9 (20%)	14 (35%)
10-<15	11 (24%)	8 (20%)
≥15	18 (39%)	12 (30%)
median (IQR)	13 (7 to 20)	10 (7 to 19)
range	1 - 46	3 - 43
BMI (for age>20) (kg/m^2)	N=24	N=21
18-<25	6 (25%)	4 (19%)
25-<30	12 (50%)	13 (62%)
≥30	6 (25%)	4 (19%)
mean ± SD	28 ± 4	27 ± 3
range	22 - 40	20 - 32
BMI Z Score* (for age≤20)	N=22	N=19
mean ± SD	0.70 ± 0.92	0.69 ± 0.86
Sex – Female n (%)	22 (48%)	22 (55%)
Race/Ethnicity		
White non-Hispanic	44 (96%)	38 (95%)
Black/African-American	1 (2%)	2 (5%)
Asian	1 (2%)	0 (0%)
HbA1c at Screening †		
7.5-7.9% [58-63 mmol/mol]	18 (39%)	14 (35%)
8.0%-8.4% [64-68 mmol/mol]	12 (26%)	15 (38%)
8.5%-10.0% [69-86 mmol/mol]	16 (35%)	11 (28%)
mean ± SD (%)	8.3 ± 0.6	8.2 ± 0.5
Total Daily Insulin (U/kg/day)		
mean ± SD	0.76 ± 0.25	0.69 ± 0.18
C-peptide (pmol/l) ‡		
median (IQR)	23.5 (8.0 to 33.1)	20.5 (8.0 to 33.1)
Blood Glucose (mmol/l)		
mean ± SD	11.0 ± 4.5	10.0 ± 3.9
Sever Hypoglycaemia in Last 6 Months		
None	46 (100%)	40 (100%)
CGM User		
Never	16 (35%)	10 (25%)
Rarely	20 (43%)	22 (55%)
Occasional	10 (22%)	8 (20%)
CGM Use in Past 3 Months		
Not at all	35 (76%)	33 (83%)
1-2 weeks	10 (22%)	6 (15%)
3-4 weeks	1 (2%)	1 (3%)

N (percentage), mean ± SD or median (interquartile range) values are shown

* BMI z score adjusted for age and gender based on 2000 CDC growth charts

†Local HbA1c measured at screening visit

‡Measured at non-hypoglycaemia

Table S2. Sensor glucose time in range according to subgroups.

	Change from baseline to 12 weeks				***P value for interaction*** †
	**Closed-loop**		**Control**		
	N *	*Mean ± SD*	*N* *	*Mean ± SD*	
Overall	46	12.8 ± 7.8	40	2.2 ± 5.8	
Age at enrolment					0.59
<13 years	11	14.7 ± 8.0	12	1.8 ± 5.5	
13 to 21 years	11	13.6 ± 9.4	8	4.2 ± 6.4	
≥22 years	24	11.6 ± 6.9	20	1.6 ± 5.9	
Sex					0.24
F	22	13.3 ± 8.2	22	2.8 ± 5.8	
M	24	12.3 ± 7.6	18	1.4 ± 5.9	
White Race					NA‡
Yes	44	12.8 ± 7.8	38	2.4 ± 5.8	
No	2	13.6 ± 11.7	2	-2.5 ± 2.3	
HbA1c at baseline					0.11
A: <8.5 %	36	11.0 ± 6.6	33	2.1 ± 5.6	
B: ≥8.5 %	10	19.4 ± 8.5	6	3.0 ± 7.6	

* Restricted to participants with non-missing data at both baseline and 12 weeks

† P value for interaction between treatment group and subgroup factor

‡ P value not calculated due to small sample size in non-white race

Table S3. Selected outcomes by glycated haemoglobin stratum at screening.

	Baseline		12 weeks	
	**Closed-loop**	**Control**	**Closed-loop**	**Control**
Screening HbA1c <8.5% (69 mmol/mol)	N=30	N=29	N=30	N=29
Percent of time with sensor glucose level in range				
3.9 to 10.0 mmol/l	54 ± 8	53 ± 9	65 ± 8	55 ± 9
<3.5 mmol/l	2.4 (0.9 to 3.8)	1.8 (0.8 to 3.1)	1.5 (1.0 to 2.5)	2.0 (1.2 to 3.1)
Mean glucose (mmol/l)	9.6 ± 0.8	9.7 ± 1.0	8.8 ± 0.6	9.6 ± 0.9
Glucose SD (mmol/l)	3.8 ± 0.4	3.8 ± 0.5	3.5 ± 0.5	3.8 ± 0.5
Total daily insulin (U/kg/day)	0.78 ± 0.22	0.67 ± 0.17	0.82 ± 0.24	0.69 ± 0.17
Glycated haemoglobin				
Percent	7.7 ± 0.3	7.7 ± 0.5	7.2 ± 0.5	7.6 ± 0.5
Millimoles per mole of nonglycated haemoglobin	61 ± 4	60 ± 6	56 ± 5	59 ± 6
Body weight change from screening (kg)			1.9 ± 2.2	1.7 ± 2.0
Screening HbA1c ≥8.5% (69 mmol/mol)	N=16	N=11	N=16	N=11
Percent of time with sensor glucose level in range				
3.9 to 10.0 mmol/l	50 ± 13	48 ± 9	65 ± 9	52 ± 10
<3.5 mmol/l	1.5 (0.8 to 2.3)	2.4 (0.6 to 3.9)	1.0 (0.9 to 1.7)	2.0 (0.5 to 2.8)
Mean glucose (mmol/l)	10.1 ± 1.5	10.1 ± 1.1	9.0 ± 0.8	9.8 ± 1.1
Glucose SD (mmol/l)	4.0 ± 0.7	3.9 ± 0.3	3.6 ± 0.6	3.9 ± 0.3
Total daily insulin (U/kg/day)	0.69 ± 0.20	0.78 ± 0.19	0.78 ± 0.28	0.77 ± 0.24
Glycated haemoglobin				
Percent	8.4 ± 0.8	8.2 ± 0.6	7.7 ± 0.8	8.0 ± 0.5
Millimoles per mole of nonglycated haemoglobin	68 ± 9	66 ± 7	60 ± 9	64 ± 5
Body weight change from screening (kg)			3.0 ± 2.5	0.6 ± 3.7

Note. Statistical testing not carried out to limit false discovery rates

Table S4. Selected secondary endpoints according to age subgroups.

		Closed-loop			Control			
		*N**	*Mean*	*SD*	*N**	*Mean*	*SD*	***P value for interaction†***
HbA1c change	*<13 years*	11	-0.6	0.5	11	-0.1	0.4	
	13 to 22 years	10	-0.6	0.5	8	-0.1	0.5	0.19
	≥22 years	24	-0.5	0.4	20	-0.2	0.3	
		*N**	*Median*	*IQR*	*N**	*Median*	*IQR*	
Change in %time sensor glucose<3.5 mmol/l	*<13 years*	11	0.5	(-0.5 to 1.2)	12	0.1	(-0.2 to 0.6)	
	13 to 22 years	11	-0.8	(-2.1 to 0.5)	8	-0.1	(-1.1 to 0.6)	0.26
	≥22 years	24	-0.5	(-1.3 to 0.4)	20	0.0	(-0.6 to 0.3)	

* Restricted to participants with non-missing data at both baseline and 12 weeks

† P value for interaction between treatment group and subgroup factor

Table S5. Comparison of continuous glucose monitoring use by treatment group.

	**Closed-loop (N=46)**			**Control (N=40)**			**P value**
	Median	*Q1*	*Q3*	*Median*	*Q1*	*Q3*	
% time CGM used	90	83	95	90	81	95	0.44
Average No. days/week	7.0	6.8	7.0	6.9	6.7	7.0	

	Closed-loop		***Control***	
	N	*%*	*N*	*%*
CGM use group				
3-<4 days	1	2	0	0
5-<6 days	2	4	1	3
6-<7 days	19	41	21	53
7 days	24	52	18	45
CGM use group				
<6 days	3	7	1	3
≥6 days	43	93	39	98

Table S6. Unscheduled visits/emails/phone calls by treatment group.

	Closed-loop	***Control***
	No. contacts	*No. contacts*
Contact		
Office visit	16	3
Email/phone	53	14

		**Closed-loop**		**Control**	
		No. subject	*%*	*No. subject*	*%*
Contact	*No. contacts/subject*				
Office visit	*0*	34	74	37	93
	1	9	20	3	8
	2	2	4	0	0
	3	1	2	0	0
	All	46	100	40	100
Email/phone	*No. contacts/subject*				
	0	16	35	32	80
	1	18	39	5	13
	2	4	9	1	3
	3	5	11	1	3
	4	3	7	1	3
	All	46	100	40	100

Table S7. Pump threshold suspend events in the closed-loop group (N=549 weeks from 46 subjects).

	24h		***Daytime***		***Night-time***	
	N	*%*	*N*	*%*	*N*	*%*
No. of low glucose suspend events per week						
<1 per wk	4	9	7	15	40	87
1 to 2 per wk	14	30	19	41	6	13
2 to 3 per wk	12	26	9	20	0	0
3 to 4 per wk	7	15	6	13	0	0
4 to 5 per wk	4	9	1	2	0	0
≥5 per wk	5	11	4	9	0	0
	Median	IQR	Median	IQR	Median	IQR
No. of low glucose suspend events per week in intervention	2.2	(1.5 to 3.7)	1.8	(1.1 to 3.0)	0.4	(0.2 to 0.8)

Table S8. Subjects excluded from per-protocol analysis. The per-protocol analysis was limited to subjects with sensor glucose data availability for at least 50% over the 12-week study period (both groups) and closed-loop use for at least 80% of the time when sensor glucose data were available (closed-loop group).

Reason	Closed-loop (N=46 randomized)	Control (N=40 randomized)
Less than 50% CGM data available during follow-up	1	1
Closed-loop use less than 80% when CGM data available	21	NA
Total number of participants excluded	22	1
Age <13 years	8	1
Age 13-<22 years	7	0
Age ≥22 years	7	0
Number of remaining participants for per-protocol analysis	24	39

Table S9. Per-protocol analysis results (primary outcome).

	Baseline		12 weeks		
	**Closed-loop (N=24)**	**Control (N=39)**	**Closed-loop (N=24)**	**Control (N=39)**	**P value**
Percent of time sensor glucose between 3.9-10.0 mmol/l	54 ± 10	52 ± 9	68 ± 8	54 ± 9	NA
Change from baseline	NA	NA	13.5 ± 7.6	2.3 ± 5.9	NA
Mean adjusted difference in change (closed-loop minus control)			12.0%		<0.0001
95% CI for the adjusted difference in change (closed-loop minus control)			(8.8%, 15.2%)		NA

Table S10. Per-protocol analysis results limited to subjects with sensor glucose data availability for at least 50% over the 12-week study period (both groups) and closed-loop use for at least 70% of the time when sensor glucose data were available (closed-loop group) (post-hoc analysis).

	Baseline		12 weeks		
	Closed-loop (N=39)	Control (N=39)	Closed-loop (N=39)	Control (N=39)	P value
Percent of time sensor glucose between 3.9-10.0 mmol/l	54 ± 9	52 ± 9	67 ± 7	54 ± 9	NA
Change from baseline	NA	NA	13.0 ± 7.7	2.3 ± 5.9	NA
Mean adjusted difference in change (closed-loop minus control)			11.7		<0.0001
95% CI for the adjusted difference in change (closed-loop minus control)			(8.9, 14.6)		NA

Table S11. Adverse events during 12-week closed-loop intervention and 12-week standard sensor-augmented pump therapy (control).

	**Closed-loop (N=46)**	**Control (N=40)**
Hospitalisation due to diabetic ketoacidosis	1	0
*Significant hyperglycaemia**	2	2
Ketonaemia	0	1
Respiratory tract infection	6	0
Gastroenteritis	1	2
Urinary tract infection	1	0
Shingles	1	0
Ptosis	1	0
Skin infection	1	0
Localised swelling (foot)	1	0
Syncope (not hypoglycaemia-related)	1	0

* Defined as capillary glucose > 16.7 mmol/l (300 mg/dl) and plasma ketones >0.6 mmol/l

Table S12. Protocol deviations by treatment group.

	Closed-loop	***Control***
	N	*N*
Severity - Major		
Informed consent	1	1
Severity - Minor		
Insulin pump	6	5
Reference sample processing	1	1
Visit schedule	30	33
Total	38	40
